# Luminescent sensing and imaging of oxygen: Fierce competition to the Clark electrode

**DOI:** 10.1002/bies.201500002

**Published:** 2015-06-25

**Authors:** Otto S. Wolfbeis

**Affiliations:** ^1^ Institute of Analytical Chemistry, Chemo‐ and BiosensorsUniversity of RegensburgRegensburgGermany

**Keywords:** fluorescence, imaging, luminescence, microsensor, nanosensor, oxygen, sensor

## Abstract

Luminescence‐based sensing schemes for oxygen have experienced a fast growth and are in the process of replacing the Clark electrode in many fields. Unlike electrodes, sensing is not limited to point measurements via fiber optic microsensors, but includes additional features such as planar sensing, imaging, and intracellular assays using nanosized sensor particles. In this essay, I review and discuss the essentials of (i) common solid‐state sensor approaches based on the use of luminescent indicator dyes and host polymers; (ii) fiber optic and planar sensing schemes; (iii) nanoparticle‐based intracellular sensing; and (iv) common spectroscopies. Optical sensors are also capable of multiple simultaneous sensing (such as O_2_ and temperature). Sensors for O_2_ are produced nowadays in large quantities in industry. Fields of application include sensing of O_2_ in plant and animal physiology, in clinical chemistry, in marine sciences, in the chemical industry and in process biotechnology.

## Introduction

Oxygen^1^ (O_2_) is the element that is most often associated with life 1. It forms a 21% fraction of the atmosphere, and water contains roughly 88.8 wt% of O_2_. It is also one of the most abundant elements of the Earth's crust. Men can survive about 1 month without food and 1 week without water supply, but usually not more than 10 minutes without air (i.e., O_2_). Oxygen is also an important “foodstuff.” Adult humans metabolize some 200–250 g per day. It, therefore, does not come as a surprise that oxygen is routinely quantified in all kinds of sciences including biochemistry, clinical chemistry, marine and space sciences, in biotechnology and environmental chemistry, to mention only the major fields.

Methods for the quantitative determination of the partial pressure of O_2_ (*p*O_2_) are therefore most important, and any new one has good chances to be accepted. It must be stated at this point that one has to differentiate between *p*O_2_ and *S*_O2_, a parameter that refers to the saturation (*S*) of hemoglobin with oxygen. *S*_O2_ is usually determined via pulse oximetry (using, for instance, ear clips) or via diffuse optical spectroscopy, near‐infrared imaging, or photoacoustic tomography. Both *S*_O2_ and *p*O_2_ are important clinical parameters. *S*_O2_, unlike *p*O_2_, does not play a role in areas outside clinical diagnosis and blood physiology. It is, therefore, surprising to see that the number of analytical (instrumental) methods for routine determination of *p*O_2_ is not truly large. Letting aside wet chemical methods such as the Winkler titration and irreversibly responding color tests, several instrumental methods for the determination of gaseous *p*O_2_ and dissolved O_2_ are known and will be briefly discussed in the next section. Despite these methods, some of which are less common, there is a need for a good way to measure *p*O_2_. Optical sensing and imaging based on the quenching of luminescence is now considered a true alternative to the methods described below and will be the focus of this review.

## Advantages and limitations of methods commonly used to determine pO_2_

In this section, I will briefly discuss various methods that are commonly used to determine *p*O_2_. The Clark electrode (based on *amperometry*) is most common. It consists of a platinum electrode covered with an oxygen‐permeable membrane, often made from an organic fluoropolymer. On applying a voltage of typically 0.7 V, oxygen is electrochemically reduced at the surface of the electrode. The Clark electrode suffers from disadvantages including (i) the consumption of oxygen (which can be detrimental in case of very small and/or oxygen‐deficient samples); (ii) the difficulty of acquiring oxygen tension over large areas (such as in oxygen histography); (iii) sensor needle‐induced damage to tissue; and (iv) the need for the presence of at least traces of water for the reduction reaction to occur. Typical tip diameters are in the order of 100 μm, but microelectrodes with 10‐μm tips are available as well 2. Patterned silicon‐integrated circuit sheets also have been described 3 that can be stretched to cover a relatively large tissue area.

The second class of electrochemical sensors for O_2_ relies on *conductometry*. They are widely used in cars (“λ‐probes”) and rely on a zirconia ceramic coated with a thin layer of platinum to form a solid‐state electrochemical fuel cell. Carbon monoxide, if present, is oxidized by O_2_ to form CO_2_ and thereby triggers the flow of a current. Both heated (>300 °C) and (less often) non‐heated forms are known. Obviously, this sensor does not directly sense O_2_, but rather the difference between the concentration of O_2_ in the exhaust gas and in the supplied air. Such sensors can hardly be used in other fields.

*Radioisotope techniques* rely on the emission of short‐lived radioisotopes of elements such as oxygen, nitrogen, and carbon. They provide excellent three‐dimensional, non‐invasive, full‐body imaging capabilities but are far beyond routine applications and expensive. On the other hand, they can report changes in perfusion, visualize oxygen metabolism, and indicate hypoxic (diseased) regions. Positron emission tomography (PET) relies on steady state measurement of γ‐rays emitted by ^15^O (with a half‐life of 2.4 minutes only) using a PET scanner under continuous inhalation of ^15^O_2_ gas. Because of its short half‐life, ^15^O_2_ must be piped directly from a medical cyclotron. Single photon emission computed tomography (SPECT), in turn, relies on the detection of single emitted γ‐ray photons (while PET detects pairs of γ‐rays following positron‐electron annihilation). Its spatial resolution is inferior to PET, but SPECT images are simpler to acquire. These methods are expensive, difficult to calibrate, and require a license to work with radioactive materials.

Magnetic resonance (MR) techniques for O_2_ rely on probes containing the elements ^19^F or ^1^H which are not radioactive. Oximetry based on ^19^F MR utilizes perfluorinated hydrocarbons (PFHCs) such as hexafluorobenzene as contrast agents. The relaxation rates of ^19^F in PFHCs depend on the local partial pressure of O_2_. The method is expensive, not routinely applicable and not well suited for non‐medicinal research because the PFHC has to be administered intravenously hours to days before measurement. Oximetry based on ^1^H MR utilizes siloxanes such as hexamethyldisiloxane (HMDSO) as contrast agents. The relaxation rates of ^1^H in HMDSO can be correlated to local *p*O_2_.

*Electron resonance* involves electron paramagnetic resonance (EPR) and dynamic nuclear polarization. O_2_ has a triplet electronic ground state and is therefore paramagnetic. However, it cannot be directly detected in living systems by EPR because of the broadening of its spectral lines. Therefore, exogenous paramagnetic substances such as nitroxides are used. They are not stable inside cells and quickly metabolized to hydroxylamines which are diamagnetic. The rate of this process depends on the local *p*O_2_. Nitroxide radicals can be replaced by insoluble particulate paramagnetic materials. These make EPR oximetry much more sensitive. Dynamic nuclear polarization is performed by collecting NMR images while irradiating the EPR resonance signal from the free radical under study. It is more sensitive and much more expensive.

## Optical sensors for oxygen perform better than others and pave the way to new applications

Optical sensing of oxygen has emerged as a true alternative to the methods mentioned above. Numerous techniques are known 4. Some sensors respond irreversibly (such as certain commercial color tests for visual read‐out). They rely on irreversible chromogenic reactions. Others work as absorptiometric probes with reversible but slow response. This essay is restricted to methods based on quenching of luminescence because it has become the accepted method. It gives an account of the technology as used in practice and focuses on essentials. In fact, all commercial solid‐state optical sensors for O_2_ rely on the use of luminescent probes (in a polymer host matrix) whose emission is dynamically (collisionally) quenched by triplet O_2_.

Optical sensors for oxygen are based on the integration of three systems. The first is the sensor chemistry. Essentially, this term relates to a mixture composed of materials including the indicator probe, at least one polymer, and – if needed – additives such as plasticizers, binders, and the like. The second is the optical system consisting of a light source, a (light‐guiding) optical system or a microscope, and photodetectors that range from photodiodes to various kinds of digital cameras and flatbed scanners 5, 6. The third is the electronic system that digitizes, handles, processes, and displays the data.

Optical methods are in the process of replacing Clark electrodes in many fields for a number of reasons:

- O_2_ is not consumed during measurements;
- luminescence‐based optical sensors are fully reversible;
- sensors can be designed for highly different levels of O_2_, i.e., from very low (ppb) to very high concentrations (or partial pressures);
- remote sensing is enabled by using optical fibers;
- miniaturized fiber optic sensors are available with 25‐μm tips;
- a sterilized sensor layer can be placed in a sample and remotely and non‐invasively read;
- planar sensors or nanosensors can be applied to imaging of O_2_, both over large areas and on a micrometer scale;
- multiple sensing at the same site is enabled by combining sensors for O_2_ with others (such as for temperature) and separating their signals by spectroscopic or electronic means;
- targeted sensing is enabled by using nanoparticles that can recognize their target;
- sensors work equally well for even extremely dry gases and dissolved oxygen;
- optical sensors work well even in strong electromagnetic fields, in radioactive environment, and under hostile environmental or chemical conditions.

On the other hand, the following aspects should be kept in mind:

- Sensor membranes usually are made from materials that have high solubility for oxygen. Hence, they will extract O_2_ from the sample which may lead to erroneous results in case of small sample volumes and/or very low levels of O_2_;
- artifacts may be encountered due to the depletion of the ground state of the indicator under conditions of strong photoexcitation, and – in case of low levels of O_2_ – the formation of large fractions of singlet O_2_, in particular when using metalloporphyrin probes 7. In any instance, the “consumption” rate is much smaller than the chemical consumption of O_2_ in Clark electrodes.

Features and limitations of optical sensors are compared to those of the Clark electrode in Table 1. Both kinds of sensors require two‐point calibration but optodes with one‐point calibration have recently been commercialized.

**Table 1:** Features of electrodes versus optical microsensors (“optodes”)

Feature	Amperometric electrode	Fiber optic microsensor
Sensing scheme	Chemical reduction of O_2_ at 0.7 V	Dynamic quenching of luminescence by O_2_
Disadvantages	Consumes O_2_; poor performance at very low levels of O_2_; prone to drift; interfered by H_2_S, O_3_ and Cl_2_	Interfered by chlorine but not by H_2_S; brittle fiber tip; more expensive than microelectrodes
Advantages	Well established; mechanically more robust; easily sterilized	Rapid (<5 s) response time; works well in electromagnetic fields; remote sensing over up to 100 m possible; stable after calibration

Optical sensors are produced by several companies. Table 2 shows a selection. Some offer fiber optic sensors and/or planar sensor films, others dedicated instrumentation (such as for whole blood analysis). Nanosensors are available from one company only.

**Table 2:** A selection of companies offering instrumentation based on fluorescence‐based sensors for O_2_

Company	URL	Technology and comments
Aandera	www.aanderaa.no/productsdetail.php?Oxygen‐Optodes‐2	Planar O_2_ sensor for marine applications; to be deployed in buoys; long operational lifetime; virtually no signal drift
Abcam	www.abcam.com/Extracellular‐Oxygen‐Consumption‐Assay‐ab197243.html	Extracellular O_2_ consumption assay; uses microplates coated with planar fluorescent sensors for O_2_
Becton Dickinson	www.bd.com/ds/productCenter/MT.asp	Testing for tuberculosis (*M. tuberculosis*) and other mycobacteria (BACTEC™ 9000 MB); comes in the form of a sophisticated instrument and also in the form of a test tube (BD BBL™ MGIT™) for visual read‐out
Centec	www.centec.de	Planar O_2_ sensor for use in wastewater and breweries; offers two analytical ranges (1 ppb to 2 ppm; and 30 ppb to 35 ppm)
Finesse Inc.	www.finesse.com/products/sensors/	Luminescent sensor (TruFluor™) that measures dissolved oxygen; dual sensor (for pH and dissolved oxygen) also available; both single‐shot and re‐usable sensors offered for fermentation process monitoring
Hach and Hach‐Lange	www.hach.com; www.hach‐lange.de	Luminescent probes for dissolved O_2_ and BOD; the O_2_ probe does not require a calibration for the entire 2‐year life of the sensor layer
Idexx Labs., inc.	https://www.idexx.com	Animal health care; VetStat™ optical electrolyte and blood gas analyzer
Ocean Optics Inc.	www.oceanoptics.com; http://oceanoptics.com/product/	So called *Foxy*™ probes (fiber optic based); rather larger fibers; intensity‐based
OptiMedical Syst. Inc.; a subsidiary of IDEXX Labs, Inc.	www.optimedical.com	Near‐patient blood gas analyzers (so‐called Opti line; CCA‐TS2™); also used in ambulance cars; portable; requires 40 μL of blood for six diagnostic parameters (O_2_ included)
Oxford Optronics	www.oxford‐optronix.com/	Fiber optic system (OxyLite™)
Oxysens Inc.	www.oxysense.com	O_2_ analyzers for permeation analysis, O_2_ transmission rate testing, for in‐line process monitoring and modified atmosphere packaging
Paar GmbH	www.anton‐paar.com/corp‐en/products/details/O2‐meter‐oxyqc/	Sensing dissolved O_2_ in beer, wine, soft drinks (OxyQC™); battery powered
Presens GmbH	www.presens.de	Fiber optic microsensors; planar sensors; flow‐through systems; probably the largest company; applications in biotechnology; marine science, testing gas tightness of bottles (soft drinks); in modified atmosphere packaging
PyroScience	www.pyro‐science.com	Sensors based on REDFLASH technology; product line ranges from microsensors to contactless sensors; work in gases and liquids; company also offers O_2_ sensor nanoparticles and solvent‐resistant oxygen sensor layers
Terumo Inc.	www.terumo‐cvs.com/products/	Widely used to monitor O_2_ during cardiopulmonary bypass operations (CDI^®^ Blood Parameter Monitoring System 500“); also measures pH and CO_2_

### The sensing scheme: Not quite new but quite robust

The work function of sensors based on the quenching of fluorescence is usually described by the Stern–Volmer equation. In its most simple form it reads as

(1) $$F_{0}/F=\tau /\tau_{0}=1+K_{SV}[O_{2}]$$

In this equation *F*_0_ and *F*, respectively, correspond to the luminescence intensities of a probe in the absence and presence of oxygen; *τ* and *τ*_0_ are the luminescence decay times of a probe in the absence and presence of oxygen, *K*_SV_ is the Stern–Volmer constant, and [O_2_] is the concentration of oxygen in the sample. The equation holds for both gaseous and dissolved O_2_ (DO).

The concentration of DO in a fluid is related to the partial pressure (*p*O_2_) of O_2_ above (and in) a fluid by Henry's law:

(2) $$p=k_{H}c$$

Here, *p* is the partial pressure of O_2_ above the fluid, *c* is the concentration of DO, and *k_H_* is the Henry constant that depends on solvent and temperature. The numerical value of *K*_H_ is 769.2 L/(mol atm) for water at 22 °C if pressure is given in atmospheric pressure units and concentration in mol/L. Henry's law can be used to calculate the molar or mass concentration of O_2_ in a sample such as blood or seawater that is in equilibrium with a gas, typically air. As a rule of thumb, water contains around 8 ppm (w/w) of O_2_ if equilibrated with air at ambient temperature and atmospheric pressure.

**Figure 1:**
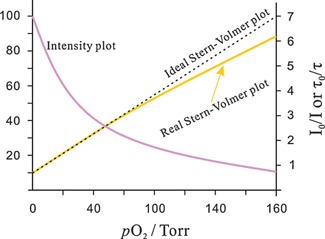
Intensity plot of the quenching of luminescence intensity by oxygen, and respective Stern–Volmer plot. The *y*‐axis on the left side gives relative intensity units. The *y*‐axis on the right refers to the Stern–Volmer plot.

Stern–Volmer (SV) plots describing the quenching of the fluorescence of indicator dyes in polymer hosts in reality are often non‐linear (see Fig. 1), mostly because of the inhomogeneous microenvironment of the dyes, but also due to the presence of void volumes in the polymer 8. In addition, the sample to be analyzed for O_2_ (such as water) may cause stronger swelling of the polymer in certain domains.

Non‐linear SV plots can be described by an extended equation 9 that uses two quenching constants (*K* ^1^_sv_ and *K* ^2^_sv_) with relative contributions of *f*_1_ and *f*_2_ (with *f*_1_ plus *f*_2_ being 1):

(3) $$\frac{F}{F_{0}}=\frac{f_{1}}{1+K_{SV}^{1}\cdot \left[O_{2}\right]}+\frac{f_{2}}{1+K_{SV}^{2}\cdot \left[O_{2}\right]}$$

The *K*_sv_ is but one useful parameter to describe the quenchability of a probe/polymer system. The *F*_0_/*F*_air_ (also termed *I*_0_/*I*_air_; the ratio of fluorescence intensities under zero oxygen and on air) are also often given to describe quenching efficiency. An *I*_0_/*I*_air_ ratio of 2, for example, indicates a system that is hardly quenched by oxygen. It will be suited to sense oxygen at relatively high pressure such as in supersonic wind tunnels. An *I*_0_/*I*_air_ ratio of >200, in turn, indicates a strongly quenchable material, well suited to determine trace O_2_, for example in almost anerobic biosystems or in deep‐sea or space research.

**Figure 2:**
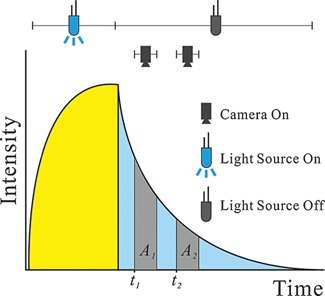
The RLD method for rapid determination of luminescence decay time. The indicator (usually a metal‐ligand complex with a relatively long decay time is first photoexcited until steady‐state fluorescence is reached. The LED is then turned off, and emission intensity is integrated over areas *A*_1_ and *A*_2_. Note that any short‐lived background luminescence is suppressed by this method.

Both fluorescence intensity and decay time (also referred to as “lifetime”^2^) can be measured. In order to work reliably, intensity‐based sensing has to be performed in the ratiometric mode. Here, an indicator dye is used along with an inert reference dye (emitting at another wavelength and not being quenched by O_2_). Decay time‐based sensing is the most robust method. It requires only a single probe, a simpler optical filter system and a single photodetector. Decay time (*τ*) is virtually independent of photobleaching and can be quickly determined via the rapid lifetime determination (RLD) method 10 as outlined in Fig. 2. The following relationship is used to calculate *τ*:

(4) $$\tau =(t_{2}-t_{1})/\ln(A_{1}/A_{2})$$

where *t*_1_ and *t*_2_ are the times at which the detector (or camera) is opened for the first and second time, respectively. Areas *A*_1_ and *A*_2_ are the luminescent intensities integrated during the time during which the detector (or camera in case of imaging) is opened.

Lifetime‐based imaging (FLIM, fluorescence lifetime imaging microscopy) has received particular attention, and methods for rapid acquisition of imaging data have been continuously improved over the years. The phasor approach represents a powerful alternative to determine fluorescence decay time 11 in that two lifetimes can be retrieved from time gating data that cannot be resolved using standard fitting. It increases the information obtained from typical measurements and simplifies analysis of FLIM data. The method of Warren et al. 12 is particularly useful. Its algorithm for global analysis of time‐correlated single photon counting or time‐gated FLIM data allows time‐resolved fluorescence data to be rapidly fit to even complex decay models.

**Figure 3:**
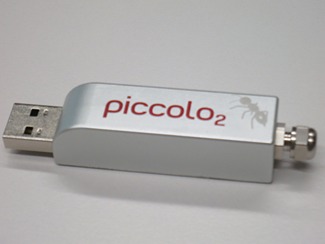
Fiber optic decay time‐based O_2_ sensor with an USB interface and (on the right side) a standard fiber connector. The fiber and its 20‐μm tip (coated with a polymer incorporating a quenchable oxygen indicator) are not shown. © PyroScience GmbH.

The use of long‐decaying indicators allows for a more simple and small optoelectronic system. Present day O_2_ sensing instrumentation based on decay time comes in sizes between those of a cigarette pack (www.presens.de) and a USB stick (www.pyro-science.com; see Fig. 3). The integration of sensing chemistry and semiconductor optoelectronics (that serve both as support and excitation light source) represents another current trend toward sensor microsystems 13.

## The proper choice of sensor materials is critical

The proper choice of the indicator dye and of the host polymer is most critical with respect to the function of an optical sensor for O_2_. The choice of these two components defines the range over which the sensor works because it governs *I*_0_/*I*_air_ values.

### Indicators possess all kinds of beautiful colors

Table 3 summarizes the indicators most often used in optical sensors for O_2_. Quaranta et al. 14 have summarized the wealth of these and other optical probes for O_2_. Many are of the metal‐ligand complex type. This is not surprising because they have long decay times which make collisional quenching by O_2_ more likely and sensing via measurement of decay times more simple. A cluster of the chemical composition Mo_6_Cl_14_, dispersed in a poly(1‐trimethylsilyl‐1‐propyne), also represents a highly attractive albeit less often used probe 15. It is used in a silica optical fiber O_2_ sensor.

**Table 3:** The most widely used fluorescent indicators for oxygen

Indicator probe	Comments
PtTFPP^a^	Porphyrin platinum(II) complex; used in various kinds of sensor formats; photostable; uncharged (resulting in good solubility in apolar polymers except silicones; decay times are in the upper μs range; excellent brightness
PdTFPP	Same as PtTFPP but with longer decay time and therefore more efficiently quenched
Ru(dpp)_3_ 2X^−^ b	Ruthenium(II) complex; used in various kinds of sensor formats; fairly photostable; cationic; this requires the presence of lipophilic counter anions (X) (such as trimethylsilyl propanesulfonate or tetrafluoroborates) in order to make them soluble in less polar polymers; if X is chloride, for example, solubility in silicones is very poor and dyes will aggregate over time; moderate brightness; decay time around 2–4 μs

All can be photoexcited with light‐emitting diodes (of various color), have large Stokes’ shifts and long decay times.

a TFPP, tetrakis(fluorophenyl) porphyrin.

b dpp, diphenylphenanthroline.

### Host polymers to carry the indicators

The host polymer is as important (in terms of analytical ranges) as the indicator 16. If polymers with high permeability for oxygen are applied, the sensor will respond to rather low O_2_ levels, while those with poor permeability will cover rather high concentrations (or partial pressures) of O_2_. Table 4 summarizes the polymers most often used in optical sensors for O_2_. The combination of an indicator with a long decay time (that favors quenching) with a polymer of very high permeability to oxygen has resulted in oxygen sensors with very low limits of detection 17.

**Table 4:** Polymer host materials often used in sensors for O_2_ (in planar or fiber optic format, or as nanobeads)

Host polymer	Comments
Silicone rubbers	Low cost; high optical purity; excellent gas permeability, good thermal and chemical stability; good adhesion to glass; very hydrophobic; poor “solvent”
Fluorosilicones	Like silicones but with even better permeability for O_2_
Polystyrene (PS) and ethyl cellulose	Low cost; easy handling; fairly good permeability for oxygen; PS displays intrinsic fluorescence under UV light; PS is often used in the form of μm‐ or nm‐sized beads
Poly(1‐trimethylsilyl‐1‐propyne) (PTMSP)	Extremely high gas permeability; expensive; prone to aging effects; often used in pressure‐sensitive paints to measure barometric pressure via *p*O_2_
Organically modified silica gels	Can be prepared from various chemical precursors; hydrophobicity and permeability are widely tunable; fluorinated ormosils have even higher permeability for O_2_; good mechanical and chemical stability

### Sensor cocktails: Easily prepared and widely applicable

Sensor materials are usually applied in the form of a “cocktail” consisting of a solution of indicator dye and a polymer (and additives if needed) in an appropriate solvent. The cocktail is then deposited in various ways, for example at the tip (distal end) of an optical fiber, in the wells of a microtiter plate, or on a planar support such as a film of poly(ethylene terephthalate). The solvent is then evaporated to leave a sensor film in thicknesses of typically 0.5–5 μm. The cocktail may also be converted into sensor nanoparticles via spray evaporation or injecting it into water. In any case, such sensor materials are fairly affordable, easily prepared, can be manufactured in large quantity, and sensors can be shaped in numerous forms.

## Optical sensing knows a variety of sensor formats

### Fiber optic sensors enable point sensing on a microscale

These represent an attractive alternative to the Clark electrode. They are intended for point measurements with a spatial resolution of typically 50 μm. The number of applications is quite large and has increased a lot in the past years 18. Such microsensors have so far experienced the largest (commercial) success and are likely to replace an estimated 60% of the current market of the Clark electrode.

### Planar sensors enable imaging and large‐area sensing

**Figure 4:**
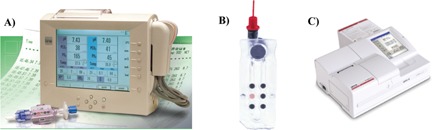
Clinical instrumentation based on planar optical sensors for oxygen. **A:** Bypass system to monitor oxygen (and pH and CO_2_) in blood that is guided through an extracorporeal system during cardiac surgery (© Terumo; www.terumo-cvs.com/). The cassette with blue ports shown at the bottom left is connected to the extracorporeal blood loop during a bypass operation, for example. It contains sensors for oxygen (and pH and CO_2_) and is optically interrogated by the instrument via fiber optic cables. **B:** Disposable microfluidic chip (six sensor spots in total) to determine O_2_ (and pH, CO_2_, Na^+^, K^+^, and chloride or glucose) in an up to 120‐μL blood sample. The disposable chip is placed under the lid of a portable analyzer (**C**) called OPTI® R Blood Gas and Electrolyte Analyzer (OptiMedical; www.optimedical.com/).

Planar sensors are used in flow systems, in microplates, diagnostic vials, and (bio) reactors where they can be placed in direct contact with the sample. Representative medical instrumentation based on planar sensors is shown and explained in Fig. 4. Another instrument based on optical O_2_ sensor technology can detect *Microbacillus tuberculosis* in whole blood (see Table 2). A 15‐mL vial (containing an oxygen‐sensitive luminescent layer at its bottom and a broth culture above it) is placed in the instrument and read out by an optoelectronic system. Sputum to be tested for *M. tuberculosis* is added to the vial. After typically 12–24 hours, a substantial fraction of O_2_ will have been consumed due to the growth of *M. tuberculosis*, and this is indicated by the luminescent O_2_ sensor on the bottom of the vial.

### Nanosensors allow us a look into cells

These types of sensor have paved the way to intracellular sensing of O_2_. Like other sensors, they consist of indicator probes in a polymer host, but usually are applied in the form of nanoparticles. Following initial work by the Kopelman group 19, various kinds of small‐particle‐based sensors have been introduced and applied to sense O_2_ inside cells or in hypoxic tumorous tissue 20, 21.

Dendrimeric nanosensors (that may also be referred to as very large molecular probes rather than being solid‐state sensors) have been reported mainly by the Vinogradov group 22. The glutamate dendrimers used (with molecular masses of typically 15–20 MDa) are good solvents for O_2_ and simultaneously serve as a shield against quenchers other than oxygen. If labeled with Pt(II)‐ and Pd(II) complexes of (benzo) porphyrins 23, they enable sensing and imaging of oxygen in the cardiovascular and cerebral systems with very short response time 24. They are commercially available under the trade name “*Oxyphor*” (www.oxygenent.net/phosphors.html).

### Imaging via sensors: Large area “Oximaging”

Two techniques are common in imaging of O_2_. One is based on the use of sensor films placed on (or under) the object of interest, the other on nanosized sensor particles (NPs). Pictures are acquired by the methods described in previous sections but also by microscopy. Sensor films (“paints”) are used, for example, to quickly image O_2_ in hypo‐oxygenated skin or cancerous tissue 25, 26, or to visualize air pressure on aircraft models in wind tunnels 27. In nanoparticle‐based approaches, oxygen‐sensitive nanoprobes are placed inside the object of interest: cells, microfluidic or vascular systems, and in tissue 28 and 3D cancer models 29. Such applications obviously require particles that are membrane permeable and not harmful to cells.

## Conclusions

Optical sensors for O_2_ have features that make them an attractive alternative to the Clark electrode. They also enable O_2_ to be sensed on a nanoscale and to be imaged over large areas. The hundreds of applications described in the past 20 years (also a result of their commercial availability) cannot be given here. Application‐oriented reviews are available (i) on applications of such sensors in biosciences, with a focus on enzymatic assays, respiration, food and microbial safety, bioreactors and fluidic chips 30; (ii) on technologies for multiple sensing of oxygen along with a second or third parameter 31; (iii) on multi‐parametric O_2_ imaging in 3D neural cell models 32; and (iv) on applications of optical O_2_ sensors in areas as diversified as blood gas and clinical analysis, imaging of O_2_ in skin and mitochondria, in toxicity testing, water research, food packaging, and in biosensors 4. Targeted sensing of O_2_ (in the extracellular and intracellular space and at mitochondria) was demonstrated 33, 34, and ultra‐sensitive optical oxygen sensors have been developed 17 for the characterization of nearly anoxic biosystems by making use of a combination of 10‐ms lifetime boron(III) and aluminum(III) chelates along with highly oxygen‐permeable perfluorinated polymers.

Planar sensors for oxygen are also used in (commercial) glucose sensors where the consumption of O_2_ caused by glucose oxidase is measured 35. In environmental sciences and water process control, optical sensors for O_2_ have been used (i) to determine biochemical O_2_ demand (BOD; see Table 2) 36, (ii) in microfluidic cell cultures 37, and (iii) in aquatic (marine) sciences 38, 39. All this is excellent proof for the power of this technology.

## Endnotes

1 The term *oxygen* is used here in place of the correct term *dioxygen* for reasons of simplicity.

2 The term *lifetime* is avoided here because it is also widely used in sensor technology in order to characterize the operational and storage lifetimes of sensors.
